# Supplementary material for: Machine learning identifies scale-free properties in disordered materials
Source: Nat Commun. 2020 Sep 24;11:4842. doi: 10.1038/s41467-020-18653-9 (PMC7519134; doi:10.1038/s41467-020-18653-9)
Supplement: Supplementary file 1 — Supplementary Information [file 41467_2020_18653_MOESM1_ESM.pdf]

Supplementary Information

Machine learning identifies scale-free properties in disordered materials

Yu et al.

Supplementary Figures

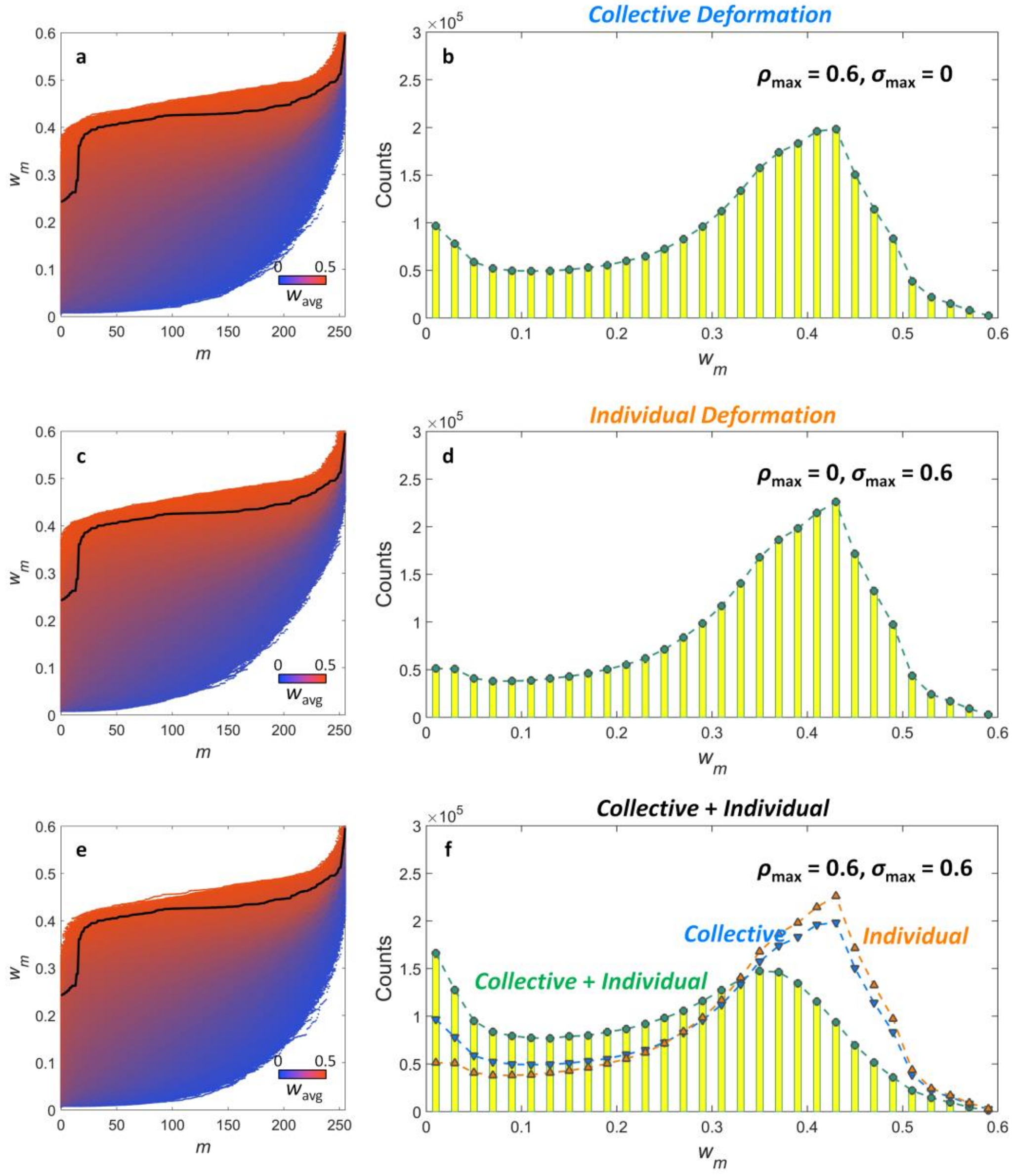

Supplementary Figure 1. Localizations with collective and individual deformations. a, b, Collective only ($\rho_{\max} = 0.6$, $\sigma_{\max} = 0$), **c, d,** individual only ($\rho_{\max} = 0$, $\sigma_{\max} = 0.6$), and **e, f,** simultaneously collective and individual deformations ($\rho_{\max} = 0.6$, $\sigma_{\max} = 0.6$). **a, c, e,** Localization values. Black solid lines denote an example realization of the localization data for each deformation condition. **b, d, f,** Statistical distributions of the localization values w_m. Blue and orange dashed lines with symbols in **f** denote the results in **b, d** for comparison. For all cases, 1×10^4 realizations are considered.

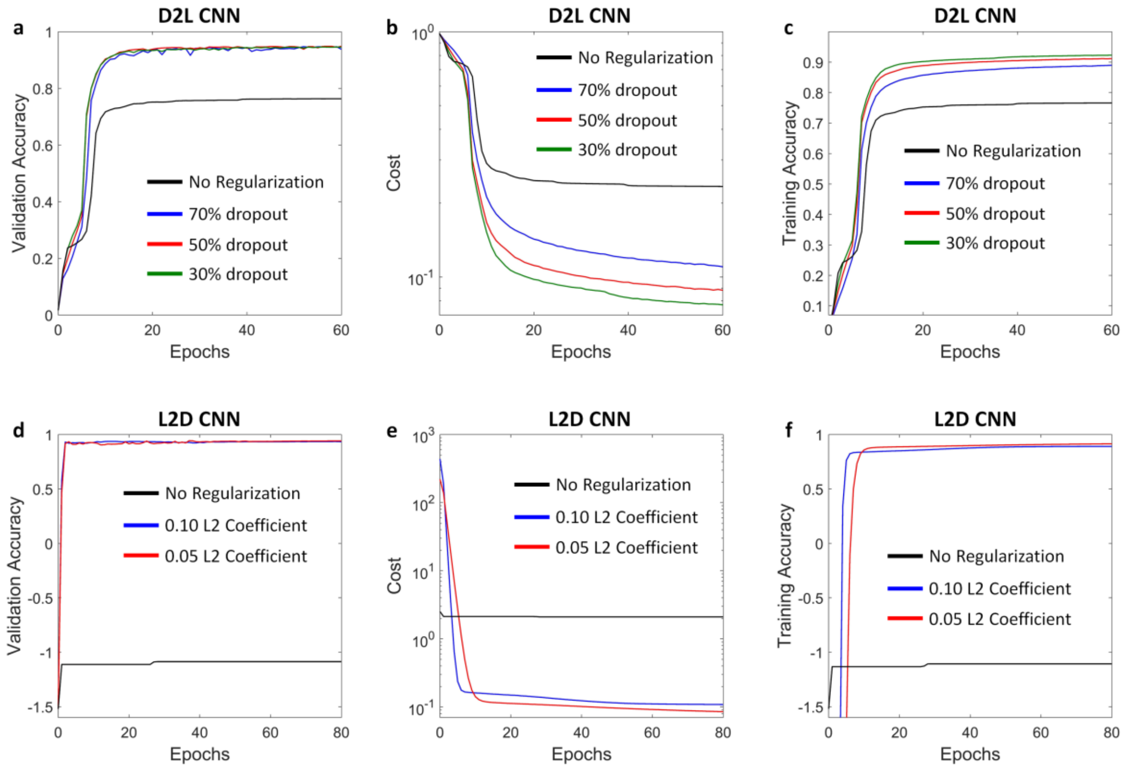

Supplementary Figure 2. Training process of convolutional neural networks. a-c, Disorder-to-localization (D2L) and **d-f,** localization-to-disorder (L2D) convolutional neural networks (CNNs) for **a, d,** validation accuracy, **b, e,** training cost functions, and **c, f,** training accuracy: **a,** $1 - L_{\mathrm{D2L}}$ for the validation dataset, **b,** L_{D2L} for the training dataset, **c,** $1 - L_{\mathrm{D2L}}$ for the training dataset, **d,** $1 - L_{\mathrm{L2D2L}}$ for the validation dataset, **e,** L_{L2D2L} for the training dataset, and **f,** $1 - L_{\mathrm{L2D2L}}$ for the training dataset. Each coloured line represents different regularization parameters for the **a-c,** dropout method and **d-f,** L2 regularization. The dropout probability in **a-c** represents the ratio of randomly assigned inactive neurons in the FC layer. A larger L2 regularization coefficient in **d-f** derives stronger regularization for the cost function in TensorFlow[1].

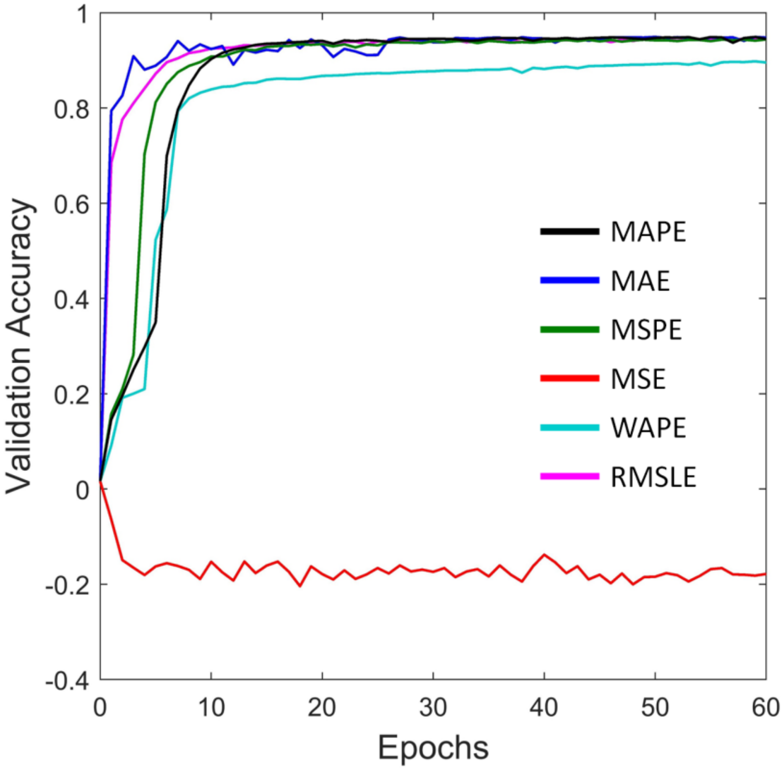

Supplementary Figure 3. Training process of the D2L CNNs with different cost functions. Validation accuracies $1 - L_{\mathrm{MAPE}}$ for the D2L CNNs trained with the MAPE, MAE, MSPE, MSE, WAPE, and RMSLE functions are evaluated. Training hyperparameters and regularization conditions are the same as those in Fig. 2 in the main text.

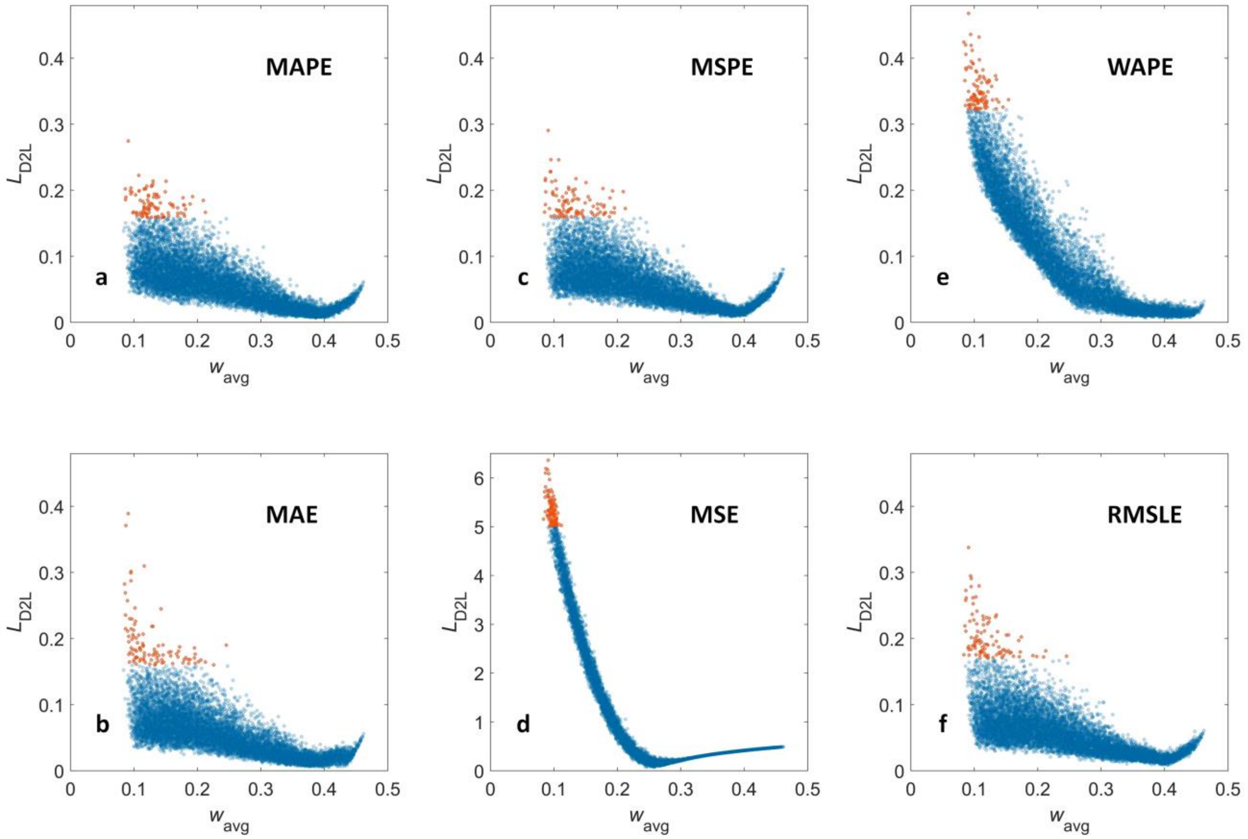

Supplementary Figure 4. Robustness of cost functions at different degrees of localization. Errors defined by $L_{\mathrm{D2L}} = L_{\mathrm{MAPE}}$ for the test dataset are evaluated for the D2L CNNs in Supplementary Figure 3, which are trained with different cost functions: **a,** MAPE, **b,** MAE, **c,** MSPE, **d,** MSE, **e,** WAPE, and **f,** RMSLE. Each dot denotes a realization of disorder having a different degree of localization (or w_{avg}). Red dots represent the realizations with errors in the top 1% value.

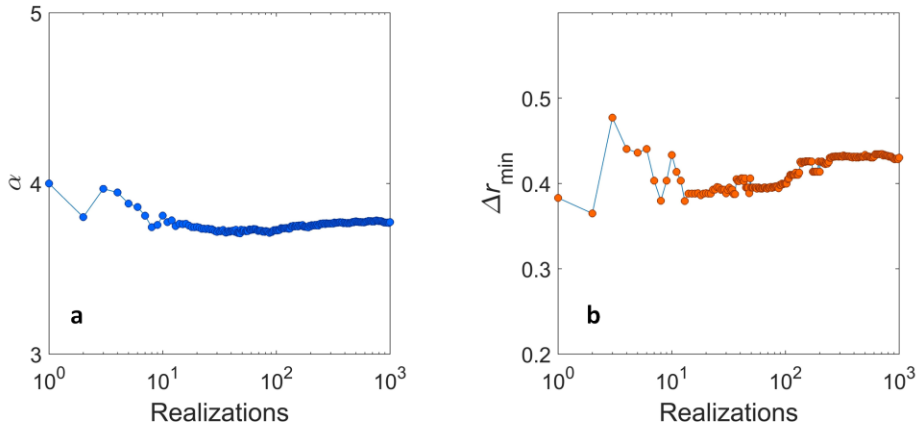

Supplementary Figure 5. Dependence of power-law parameters on the number of realizations. a, Power-law exponent α. **b,** The lower bound of the heavy tail Δr_{min}.

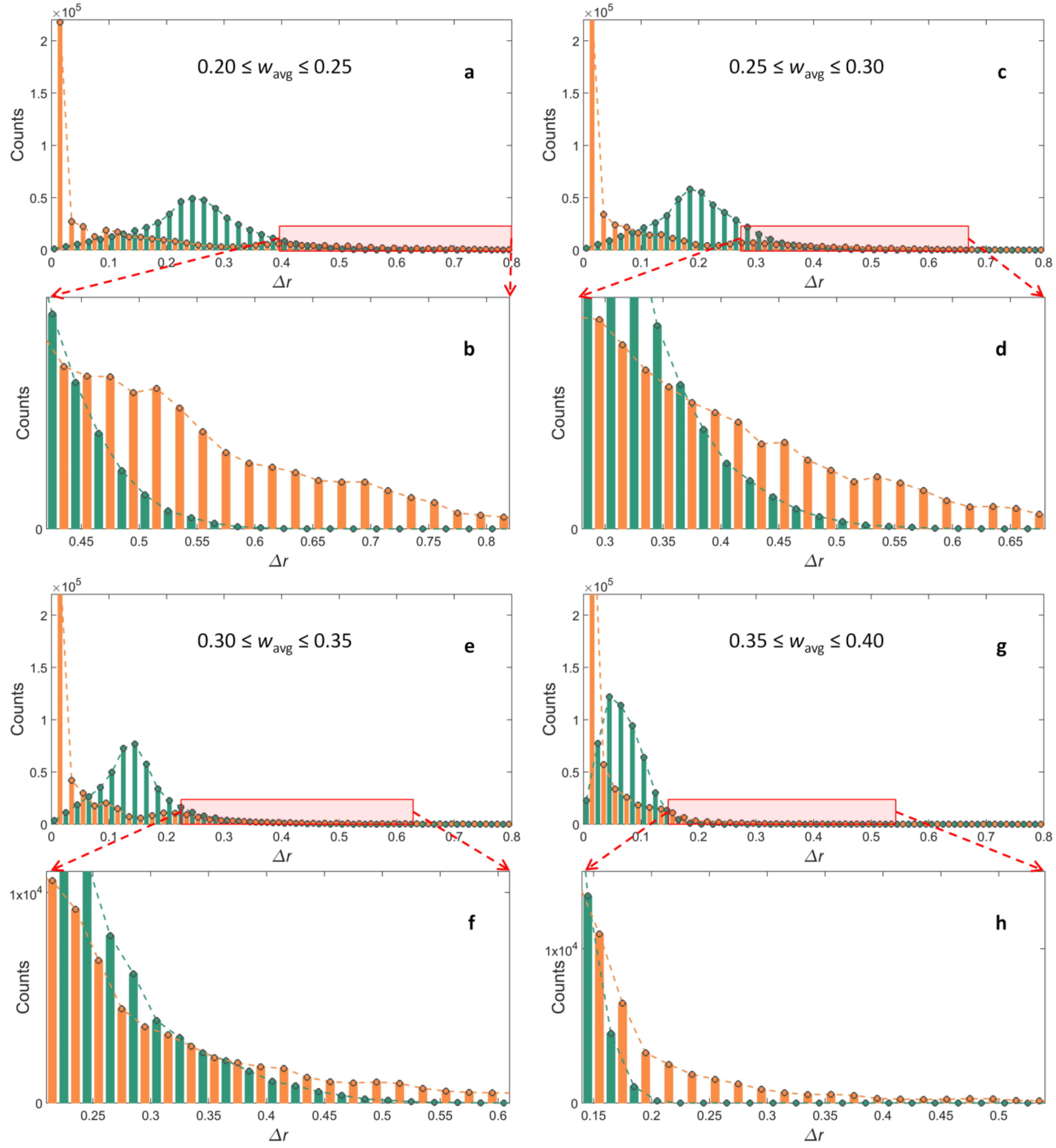

Supplementary Figure 6. Statistical distributions for different degrees of localization. The strength of the lattice deformation Δr is shown for the seed (green) and machine-learning-generated (orange) structures. **a, b,** $0.20 \leq w_{\mathrm{avg}} \leq 0.25$ (1823 realizations), **c, d,** $0.25 \leq w_{\mathrm{avg}} \leq 0.30$ (1377 realizations), **e, f,** $0.30 \leq w_{\mathrm{avg}} \leq 0.35$ (1588 realizations), and **g, h,** $0.35 \leq w_{\mathrm{avg}} \leq 0.40$ (2123 realizations) in the ML design. The plots in **b**, **d**, **f**, and **h** show the extended plot near a heavy tail in **a**, **c**, **e**, and **g**, respectively.

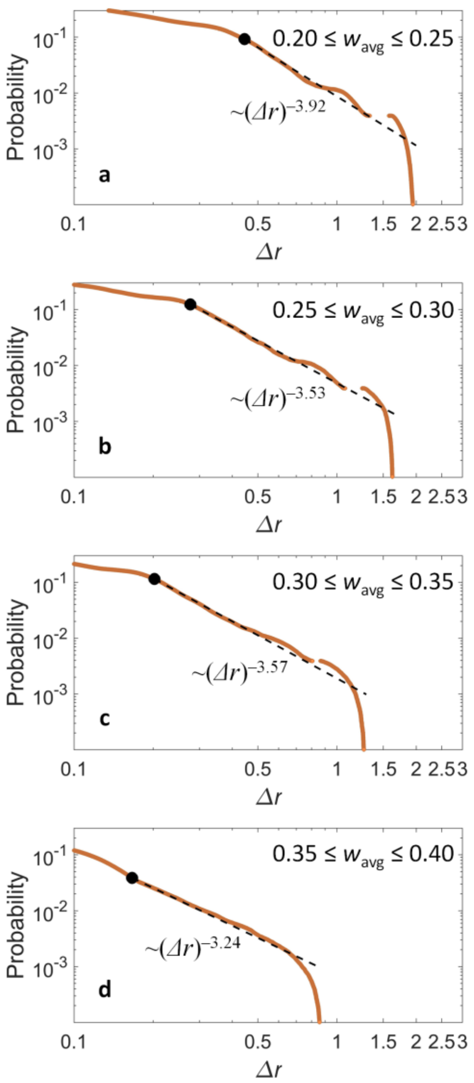

Supplementary Figure 7. Power-law analysis near heavy tails. Each plot shows the log-log plot of each case in Supplementary Figure 6, representing the power-law distribution. The orange line (composed of discretized points) represents the complementary cumulative distribution function (CDF) obtained from the data set in Supplementary Figure 6. The black dashed line represents the best power-law fit to the data using the method in Supplementary References 2,3. The black dot represents the lower bound $\Delta r_{\min}$ to the power-law behaviour. **a,** $0.20 \leq w_{avg} \leq 0.25$ (1823 realizations), **b,** $0.25 \leq w_{avg} \leq 0.30$ (1377 realizations), **c,** $0.30 \leq w_{avg} \leq 0.35$ (1588 realizations), and **d,** $0.35 \leq w_{avg} \leq 0.40$ (2123 realizations) in the machine learning design.

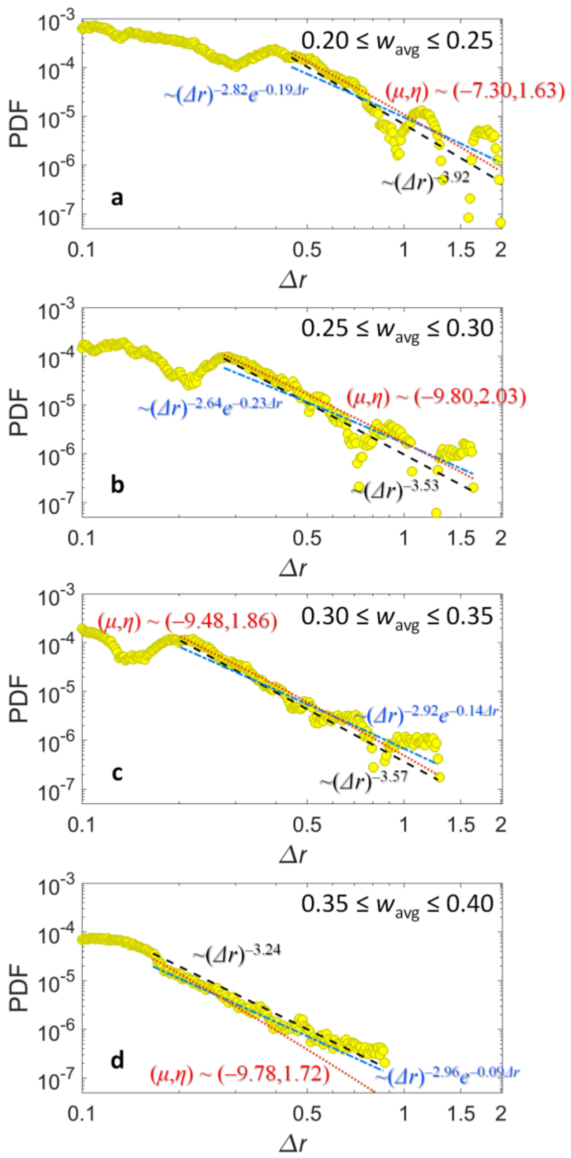

Supplementary Figure 8. Fitting with other heavy-tailed distributions. a, $0.20 \leq w_{avg} \leq 0.25$ (1823 realizations), **b,** $0.25 \leq w_{avg} \leq 0.30$ (1377 realizations), **c,** $0.30 \leq w_{avg} \leq 0.35$ (1588 realizations), and **d,** $0.35 \leq w_{avg} \leq 0.40$ (2123 realizations) in the machine learning design. The yellow line (composed of discretized points) represents the probability density function (PDF) obtained from the data set in Supplementary Figure 6. The black dashed line represents the best power-law fit to the data using the method in Supplementary References 2,3, which is the PDF representation of the black dashed line in Supplementary Figure 7. Blue and red lines represent the fitting with a power-law distribution with an exponential cutoff and a log-normal distribution, respectively.

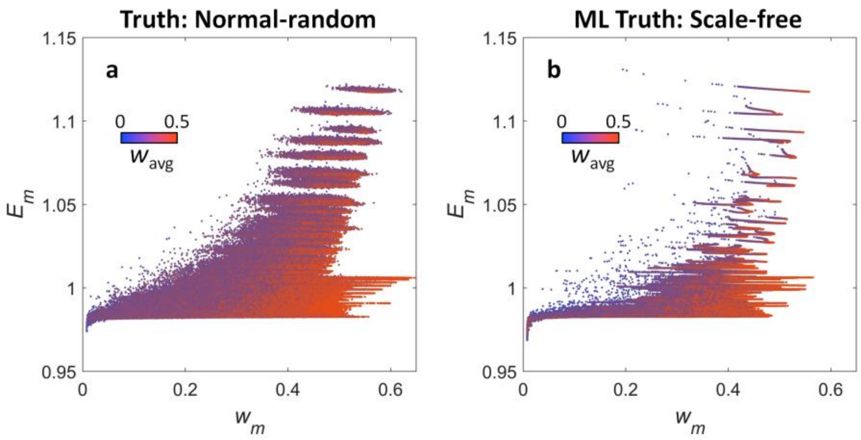

Supplementary Figure 9. Energy spectra of disordered structures with different microstructural statistics. Localization-energy relations of **a,** normal-random disordered structures obtained from Eq. (5) in Methods and **b,** scale-free disordered structures obtained from the localization-to-disorder (L2D) convolutional neural network.

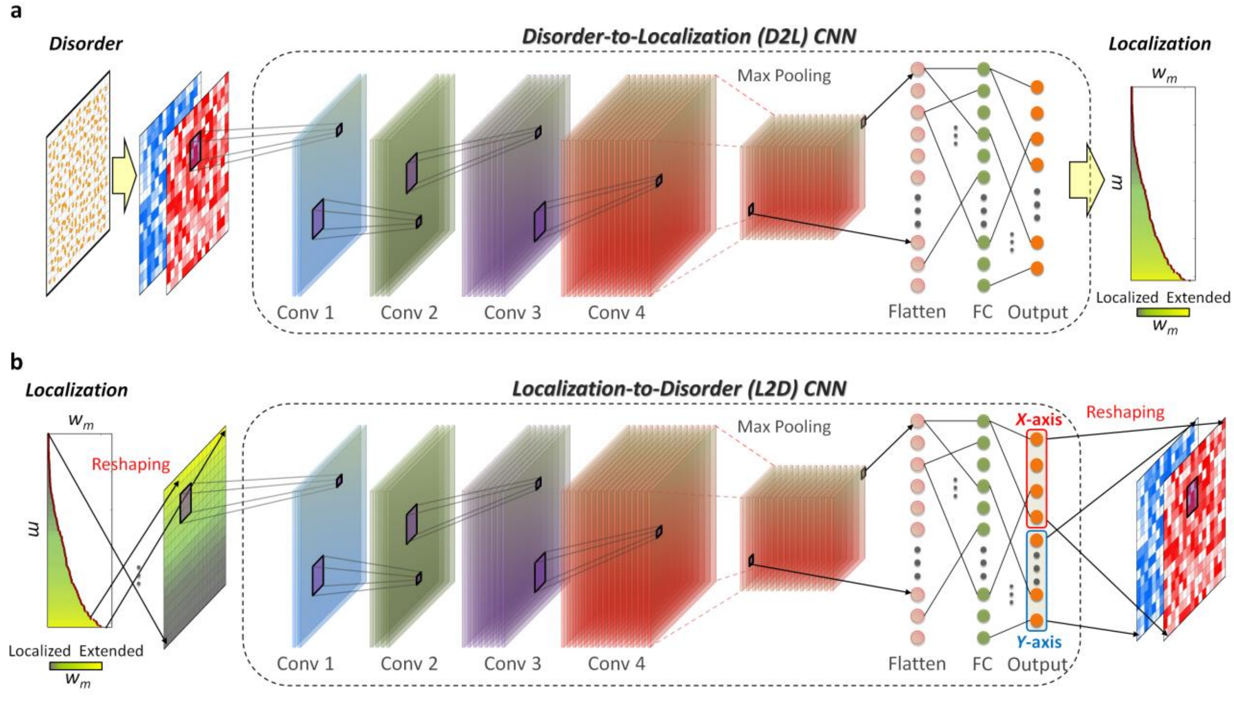

Supplementary Figure 10. Machine learning network structures with a single pooling layer. **a,** Disorder-to-localization (D2L) convolutional neural network (CNN). **b,** Localization-to-disorder (L2D) CNN.

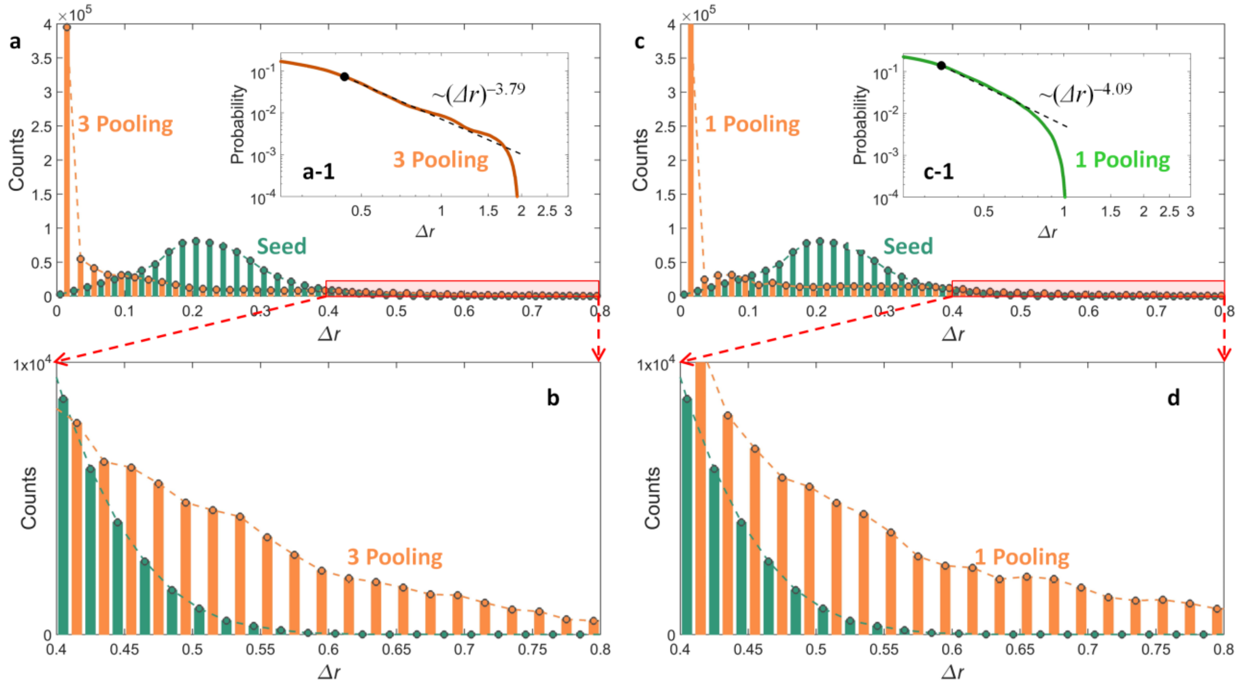

Supplementary Figure 11. Heavy tails in different machine learning architectures. Statistical distributions of the strength of the lattice deformation Δr in the seed (green) and machine-learning- (ML-) generated (orange) structures for 3200 realizations satisfying $0.20 \leq w_{avg} \leq 0.30$ in the 3-pooling-layer ML design: **a, b,** the distribution of the 3-pooling-layer design, which is Fig. 4g in the main text and is shown for comparison and **c, d,** the distribution of the 1-pooling-layer design. The figures in **b** and **d** show the extended plot of those in **a** and **c** near the heavy tails. The insets **a-1** and **c-1** show the log-log plot. The orange and green lines (composed of discretized points) represents the complementary cumulative distribution function (CDF) obtained from the data sets in **a** and **c**. The black dashed lines represent the best fit to the data using the method in Supplementary References 2,3. The black dot represents the lower bound to the power-law behaviour.

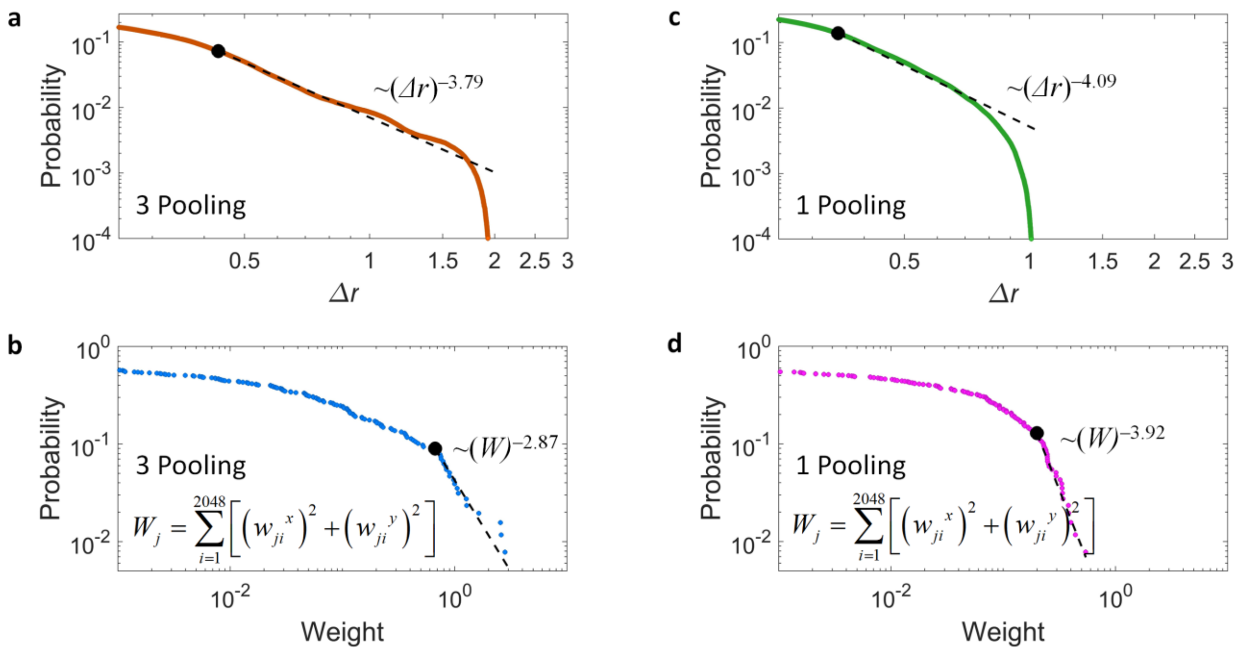

Supplementary Figure 12. Relationships between disordered materials and machine learning networks for different architectures. **a, b,** 3-pooling-layer machine learning (ML) design and **c, d,** 1-pooling-layer ML design. **a, c,** Power-law fitting of the statistical distribution of Δr in ML-generated disordered structures. **b, d,** Power-law fitting of the statistical distribution of the weight parameter W_j. **a** and **b** are Figs. 5a and 5b in the main text, respectively, and are shown for comparison.

Supplementary Notes

Supplementary Note 1. Training datasets from collective and individual deformations

To avoid overfitting in machine learning (ML), it is helpful to prepare a good training dataset that includes representative examples for each class of features. Therefore, for the inference of the relationship between wave localization and disordered structures, we need to prepare a dataset that covers the maximum range of microstructural patterns and wave localization values.

For this purpose, we compare the collective and individual deformations of atomic sites, as discussed in the Methods section. First, $\rho_{\max} \neq 0$ and $\sigma_{\max} = 0$ results in the collective deformation: the same amount of perturbation for all atoms, while the randomly assigned azimuthal angle $u_i(0, 2\pi)$ to the ith atom leads to disordered structures with broken discrete translational symmetry, and $\rho = \rho_{\max}u(0, 1)$ leads to different levels of perturbation for each realization. This collective deformation provides rigorously homogeneous patterns of perturbation strength. On the other hand, $\rho_{\max} = 0$ and $\sigma_{\max} \neq 0$ results in the individual deformation: randomly assigned perturbation for each atom, which has an average perturbation of $\sigma = \sigma_{\max}u(0, 1)$ for each spatial axis. This individual deformation provides locally inhomogeneous but statistically homogeneous perturbation strength[4]. Supplementary Figure 1a-d shows the localization and its statistics for collective (Supplementary Figures 1a and 1b) and individual (Supplementary Figures 1c and 1d) deformations. Although localization properties are similar in both deformations, their microstructural patterns of perturbation strength are different: collective patterns, which are homogeneous in all length scales, and individual patterns, which are locally inhomogeneous and statistically homogeneous. To cover the mixing of collective and individual deformations, we set the condition of $\rho_{\max} \neq 0$ and $\sigma_{\max} \neq 0$ (Supplementary Figures 1e and 1f). The combination of collective and individual deformations provides more equally

distributed localization values for the dataset (dashed lines in Supplementary Figure 1f), which prevents overfitting to certain values of localization.

Supplementary Note 2. Training process and cost functions

Supplementary Figure 2 shows the training process of the disorder-to-localization (D2L) and localization-to-disorder (L2D) convolutional neural networks (CNNs). The validation accuracy (Supplementary Figure 2a,d) is calculated by applying the validation dataset to the CNNs after the training of each epoch. The cost function (Supplementary Figure 2b,e) is evaluated with the training dataset during the optimization process. The training accuracy (Supplementary Figure 2c,f) is simply derived from the obtained cost function, as $1 - L_{\mathrm{D2L}}$ for the D2L CNN and $1 - L_{\mathrm{L2D2L}}$ for the L2D CNN.

The dropout method[5] operates better than the L2 regularization[6] in the training of the D2L CNN, and the L2 regularization provides excellent training performance for the L2D CNN, which actually entails training the localization-to-disorder-to-localization (L2D2L) CNN with the fixed D2L CNN part. Because we apply the dropout method and L2 regularization to the D2L and L2D CNNs, respectively, each CNN during the training process does not exhibit the complete representation ability when evaluating the cost function and training accuracy. This degraded representation ability is recovered after the training of each epoch (or when evaluating the validation or test accuracy). Therefore, the validation and test accuracies are slightly higher than the training accuracy at the same epoch.

When training the CNNs, we employ the mean absolute percentage error (MAPE) cost function[7,8] (Eq. (3) in the main text, setting $L_{\mathrm{MAPE}} = L_{\mathrm{D2L}}$ for the discussion in this Supplementary Note). However, we can also consider various options for the cost function in the regression problem between wave localization and disordered structures. To explore the conditions of the cost function, we further examine the following cost functions for the training process of the D2L CNN, in addition to the MAPE cost function L_{MAPE}:

mean absolute error (MAE) cost function[9]

$$L_{\mathrm{MAE}} = \sum_{m=0}^{N-1} \left| w_m^{\mathrm{True}} - w_m^{\mathrm{ML}} \right|, \qquad \text{(Supplementary Equation 1)}$$

mean-squared percentage error (MSPE) cost function[10]

$$L_{\mathrm{MSPE}} = \sum_{m=0}^{N-1} \left| \frac{w_m^{\mathrm{True}} - w_m^{\mathrm{ML}}}{w_m^{\mathrm{True}}} \right|^2, \qquad \text{(Supplementary Equation 2)}$$

mean-squared error (MSE) cost function[11]

$$L_{\mathrm{MSE}} = \sum_{m=0}^{N-1} \left| w_m^{\mathrm{True}} - w_m^{\mathrm{ML}} \right|^2, \qquad \text{(Supplementary Equation 3)}$$

weighted absolute percentage error (WAPE) cost function[12]

$$L_{\mathrm{WAPE}} = \frac{\sum_{m=0}^{N-1} \left| w_m^{\mathrm{True}} - w_m^{\mathrm{ML}} \right|}{\sum_{m=0}^{N-1} w_m^{\mathrm{True}}}, \qquad \text{(Supplementary Equation 4)}$$

and root-mean-squared logarithmic error (RMSLE) cost function[13]

$$L_{\mathrm{RMSLE}} = \sqrt{\sum_{m=0}^{N-1} \left| \log\left(w_m^{\mathrm{True}} + 1\right) - \log\left(w_m^{\mathrm{ML}} + 1\right) \right|^2}. \qquad \text{(Supplementary Equation 5)}$$

Notably, only the MAPE and MSPE cost functions provide the normalized error for the degree of localization (or the value of w_m^{True}).

Supplementary Figure 3 shows the validation accuracy of each D2L CNN trained with the above cost functions. The validation accuracy is evaluated with L_{MAPE} to make a fair comparison. At the same training hyperparameters and regularization method, the MAPE, MAE, MSPE, and RMSLE functions result in similar validation accuracies, which are superior to those of the MSE and WAPE functions. Notably, the MSE function does not lead to the convergence to

a high accuracy value. This originates from the functional form in Supplementary Equation 3, which magnifies the error of outliers, here, the data at the regime of strong localization; small w_m is more sensitive to numerical errors than large w_m.

The robustness of cost functions to outliers can be evaluated by measuring the test accuracy of each realization, which is in the specific regime of localization defined by the average mode area $w_{\mathrm{avg}} = \sum_{m=1}^{N} w_m / N$. Strong disorder with a small value of w_{avg} results in the sensitive response to errors $w_m^{\mathrm{True}} - w_m^{\mathrm{ML}}$, easily leading to outliers in the test accuracy. Supplementary Figure 4 shows the errors calculated with the test dataset, for the D2L CNNs trained in Supplementary Figure 3 with different cost functions. For each realization, we evaluate an error with $L_{\mathrm{D2L}} = L_{\mathrm{MAPE}}$ to make a fair comparison. In every case, an error is more significant for smaller w_{avg}, which denotes the regime of strong disorder. The cases of MSE and WAPE functions result in substantial errors as expected in Supplementary Figure 3. Notably, although the other cost functions (MAPE, MAE, MSPE, and RMSLE) represent higher and similar accuracies in Supplementary Figure 3, the normalized cases of the MAPE and MSPE functions are more robust than the MAE and RMSLE functions, with the decreased outliers in the regime of strong disorder (red dots near $0.1 \leq w_{\mathrm{avg}} \leq 0.2$). Therefore, the MAPE and MSPE functions are proper to handle the regression between localization and disorder.

Supplementary Note 3. Dependence of power-law distributions on data size

To examine the reliability of the power-law fitting result in the main text, we analyse the data-size dependence of the power-law exponent α for $(\Delta r)^{-\alpha}$ and the lower bound of the heavy tail Δr_{min}. The data size is determined by the number of realizations, where each realization denotes an ML-generated disordered structure composed of 256 atoms. Supplementary Figures 5a and 5b represent α and Δr_{min} as functions of the realization number, respectively. The result shows that the power-law exponent α is saturated with ~10^1 (2560 atoms) realizations, while the lower bound of the heavy tail Δr_{min} is saturated with ~10^2 (25600 atoms) realizations. Notably, even a single realization also has a similar value of α and Δr_{min}.

Supplementary Note 4. Scale invariance for different degrees of localization

In Fig. 4 in the main text, we analyse the microstructural statistics of ML-generated disordered structures, which have an average mode area w_{avg} in the range of $0.20 \leq w_{avg} \leq 0.30$. In this Supplementary Note, we compare the microstructural statistics of ML-generated structures in different degrees of localization to examine whether the scale invariance is universally observed. Supplementary Figure 6 shows the microstructural statistics of the seed and ML-generated structures for different ranges of w_{avg}. The range of statistical distributions decreases for weaker localization (or larger w_{avg}), showing the convergence to unperturbed crystals with a maximum w_{avg}. However, a "heavy-tail" distribution of ML-generated structures is always maintained regardless of the degrees of localization, especially when compared to a normal distribution of seed structures.

In Supplementary Figure 7, we also apply the analysis based on the maximum-likelihood fitting method with goodness-of-fit tests[2,3] to each case of statistical distributions. Regardless of the degrees of localization, the ML-generated structures present a very similar statistical distribution near its "heavy tail", providing the power-law distribution $(\Delta r)^{-\alpha}$ where $3.24 \leq \alpha \leq 3.92$. Therefore, the scale invariance of ML-generated disordered structures is universally observed for varying degrees of localization, although the position of heavy tails changes to meet the target localization condition.

Supplementary Note 5. Fitting with other heavy-tailed distributions

In the main text, we show the fitting of ML-generated microstructural statistics with a power-law distribution $(\Delta r)^{-\alpha}$. There also exist various options for the fitting of heavy-tailed distributions, mainly appearing to be between power-law and exponential distributions[2,14]. Among several possible choices, in this Note, we examine two different heavy-tailed distributions:

power-law distribution with an exponential cutoff

$$p(\Delta r) = (\Delta r)^{-\alpha} e^{-\gamma \Delta r}, \qquad \text{(Supplementary Equation 6)}$$

and log-normal distribution

$$p(\Delta r) \sim \frac{1}{\eta \Delta r \sqrt{2\pi}} \exp\left[-\frac{(\log \Delta r - \mu)^2}{2\eta^2} \right], \qquad \text{(Supplementary Equation 7)}$$

where $p(\Delta r)$ denotes the probability density function (PDF). By fitting the PDF of ML-generated microstructural statistics with Supplementary Equations 6 and 7, we obtain the critical parameters (α, γ) for the power-law distribution with an exponential cutoff and (μ, η) for the log-normal distribution.

In Supplementary Figure 8, the fitting results (blue and red dotted lines) are compared with the power-law fitting in Supplementary Figure 7 (black dashed lines). First, although every distribution provides reasonable and comparable fitting results, almost linear responses in the log-log plot—the signature of the scale invariance—are consistently observed in the range of $\Delta r \leq 1$. This scale-free property is apparent when comparing the power-law distributions without or with an exponential cutoff. From the definition of Supplementary Equation 6, the condition of $\Delta r \leq 1$ exhibits the power-law-dominant behaviour due to $\gamma \Delta r \ll 1$. Notably, weak disorder (Supplementary Figure 8c,d with $\gamma = 0.14$ and 0.09) provides more power-law-dominant behaviours than those of strong disorder (Supplementary Figure 8a,b with $\gamma = 0.19$ and 0.23).

Supplementary Note 6. Energy spectra in normal-random and scale-free disorder

Supplementary Figure 9 shows the energy spectra with respect to localization values in normal-random (Supplementary Figure 9a) and scale-free (Supplementary Figure 9b) disordered structures. The set of structures is composed of 5057 different realizations, which achieve the test accuracy over 80% between the target localization from normal-random disordered structures and the true values from ML-generated scale-free disordered structures. Despite very similar localization values (Figs. 3c and 3e in the main text), normal-random and scale-free disordered structures exhibit apparently different energy spectra, wherein there are more spectrally discrete states in scale-free disordered structures. Thus, the proposed ML inverse design method provides the realization of material structures for the target property of "selected" wave quantities (in this work, localization). Depending on the setting of input and output data of the CNNs, the result of wave localization can be extended into the independent and systematic handling of other wave quantities, such as bandgap materials with different localization properties[15] and broadband angular scattering with designed spectral responses[16].

Supplementary Note 7. Scale invariance in a different ML architecture

To examine the generality of scale invariance observed in the main text, we conduct an ablation study by investigating another ML architecture: a single pooling layer design. The new architecture, having a comparable number of parameters with that of the original one (roughly 1.8×10^7 parameters each in the D2L and L2D CNNs), is shown in Supplementary Figure 10. For both D2L and L2D CNNs, the numbers of filters of the convolution layers are set to 16, 32, 64, and 128 in the first, second, third, and fourth layers, respectively. A max pooling layer is used after the fourth convolution layer. The reshaped 1D array is then connected to the FC layer, which has 2048 neurons. We apply the dropout method[5] in the D2L CNN (keeping 60% of FC neurons) and apply the L2 regularization[6] in the L2D CNN (scale parameter: 0.02). All the other conditions are the same as those of the original 3-pooling-layer design. The D2L and L2D2L CNNs achieve the test accuracies roughly 94.89% and 95.06%, respectively. We compare the target localizations to the Hamiltonian-calculated true values of ML-generated disordered structures, achieving the good agreement (roughly 84.32%).

Supplementary Figure 11 compares the microstructural statistics of the seed and ML-generated structures in the original design (Supplementary Figure 11a,b, which is the same as Fig. 4g,h in the main text) and the new design (Supplementary Figure 11c,d). In both cases, the ML-generated class follows power-law statistics (inset (a-1) and (c-1) each of Supplementary Figure 11a,c) and possesses a "heavy-tail" distribution (Supplementary Figure 11b,d). However, the shape and range of the "tail" are dependent on the ML architecture with different power-law fitting results: $\sim(\Delta r)^{-3.79}$ in the 3-pooling-layer ML and $\sim(\Delta r)^{-4.09}$ in the 1-pooling-layer ML.

Supplementary Figure 12 represents the comparison of the relationships between ML architectures and ML-generated disordered structures in the 3-pooling-layer (Supplementary

Figure 12a,b) and 1-pooling-layer designs (Supplementary Figure 12c,d). The quantity $W_j = \sum_i[(w_{ji}^x)^2 + (w_{ji}^y)^2]$ for the weight strengths from the FC layer (2048 neurons) to the output layer (512 neurons) is used to estimate the critical weights in the ML architecture. We note that while the heavy-tailed distribution is always maintained, the decrease of the tail length in the 1-pooling-layer design (Supplementary Figure 12c versus Supplementary Figure 12a) originates from the decreased range of the weight strength W_j (Supplementary Figure 12d versus Supplementary Figure 12b), which shows that the control of the W_j distribution (or ML architecture) leads to the corresponding alteration of ML-generated structures.

Supplementary References

1 Abadi, M. *et al.* Tensorflow: Large-scale machine learning on heterogeneous distributed systems. *Preprint at* http://arxiv.org/abs/1603.04467 (2016).

2 Clauset, A., Shalizi, C. R. & Newman, M. E. Power-law distributions in empirical data. *SIAM Rev.* **51**, 661-703 (2009).

3 Alstott, J. & Bullmore, D. P. powerlaw: a Python package for analysis of heavy-tailed distributions. *PLoS One* **9** (2014).

4 Torquato, S. *Random heterogeneous materials: microstructure and macroscopic properties*. Vol. 16 (Springer Science & Business Media, 2002).

5 Srivastava, N., Hinton, G., Krizhevsky, A., Sutskever, I. & Salakhutdinov, R. Dropout: a simple way to prevent neural networks from overfitting. *J. Mach. Learn. Res.* **15**, 1929-1958 (2014).

6 Goodfellow, I., Bengio, Y. & Courville, A. *Deep learning*. (MIT press, 2016).

7 Yu, R., Li, Y., Shahabi, C., Demiryurek, U. & Liu, Y. *Deep learning: A generic approach for extreme condition traffic forecasting*. In *Proceedings of the 2017 SIAM international Conference on Data Mining* 777-785 (2017).

8 Yildiz, B., Bilbao, J. I. & Sproul, A. B. A review and analysis of regression and machine learning models on commercial building electricity load forecasting. *Renew. Sust. Energ. Rev.* **73**, 1104-1122 (2017).

9 Willmott, C. J. & Matsuura, K. Advantages of the mean absolute error (MAE) over the root mean square error (RMSE) in assessing average model performance. *Clim. Res.* **30**, 79-82 (2005).

10 Zell, J., Bösch, B. & Kändler, G. Estimating above-ground biomass of trees: comparing Bayesian calibration with regression technique. *Eur. J. For. Res.* **133**, 649-660 (2014).

11 Wackerly, D., Mendenhall, W. & Scheaffer, R. L. *Mathematical statistics with applications*. (Cengage Learning, 2014).

12 Louhichi, K., Jacquet, F. & Butault, J. P. Estimating input allocation from heterogeneous data sources: A comparison of alternative estimation approaches. *Agric. Econ. Res. Rev.* **13**, 83-102 (2012).

13 Chen, P.-C. *et al.* Predicting station level demand in a bike-sharing system using recurrent neural networks. *IET Intell. Transp. Sy.* **14**, 554-561 (2020).

14 Barabási, A.-L. *Network science*. (Cambridge university press, 2016).

15 Yu, S., Piao, X., Hong, J. & Park, N. Bloch-like waves in random-walk potentials based on supersymmetry. *Nat. Commun.* **6**, 8269 (2015).

16 Chung, K. *et al.* Flexible, Angle-Independent, Structural Color Reflectors Inspired by Morpho Butterfly Wings. *Adv. Mater.* **24**, 2375-2379 (2012).
